# Quantification of Chitinase mRNA Levels in Human and Mouse Tissues by Real-Time PCR: Species-Specific Expression of Acidic Mammalian Chitinase in Stomach Tissues

**DOI:** 10.1371/journal.pone.0067399

**Published:** 2013-06-27

**Authors:** Misa Ohno, Yuto Togashi, Kyoko Tsuda, Kazuaki Okawa, Minori Kamaya, Masayoshi Sakaguchi, Yasusato Sugahara, Fumitaka Oyama

**Affiliations:** 1 Department of Applied Chemistry, Kogakuin University, Hachioji, Tokyo, Japan; 2 Department of Environmental and Energy Chemistry, Kogakuin University, Hachioji, Tokyo, Japan; Massachusetts General Hospital, United States of America

## Abstract

Chitinase hydrolyzes chitin, which is an *N*-acetyl-D-glucosamine polymer that is present in a wide range of organisms, including insects, parasites and fungi. Although mammals do not contain any endogenous chitin, humans and mice express two active chitinases, chitotriosidase (Chit1) and acidic mammalian chitinase (AMCase). Because the level of expression of these chitinases is increased in many inflammatory conditions, including Gaucher disease and mouse models of asthma, both chitinases may play important roles in the pathophysiologies of these and other diseases. We recently established a quantitative PCR system using a single standard DNA and showed that AMCase mRNA is synthesized at extraordinarily high levels in mouse stomach tissues. In this study, we applied this methodology to the quantification of chitinase mRNAs in human tissues and found that both chitinase mRNAs were widely expressed in normal human tissues. Chit1 mRNA was highly expressed in the human lung, whereas AMCase mRNA was not overexpressed in normal human stomach tissues. The levels of these mRNAs in human tissues were significantly lower than the levels of housekeeping genes. Because the AMCase expression levels were quite different between the human and mouse stomach tissues, we developed a quantitative PCR system to compare the mRNA levels between human and mouse tissues using a human-mouse hybrid standard DNA. Our analysis showed that Chit1 mRNA is expressed at similar levels in normal human and mouse lung. In contrast, the AMCase expression level in human stomach was significantly lower than that expression level observed in mouse stomach. These mRNA differences between human and mouse stomach tissues were reflecting differences in the chitinolytic activities and levels of protein expression. Thus, the expression level of the AMCase in the stomach is species-specific.

## Introduction

Chitin, which is a polymer of *N*-acetyl-D-glucosamine, is the second most abundant polysaccharide in nature [1]. It is an integral component of the exoskeletons of crustaceans and insects, the microfilarial sheaths of parasitic nematodes and fungal cell walls [1], [2].

Chitinases are enzymes that digest the chitin polymer. Although mammals do not produce chitin, humans and mice have two genes that encode active chitinases, chitotriosidase (Chit1) and acidic mammalian chitinase (AMCase) [2], [3]. Chit1 was the first mammalian chitinase to be purified and cloned [4], [5]. AMCase, which is the second most active chitinase in mammals, was identified as a compensatory enzyme for Chit1 and named for its optimal activity in acidic conditions [6].

Both enzymes exhibit sequence homology to bacterial chitinases and belong to family 18 of glycosyl hydrolases, which also includes chitinase-like proteins that are structurally related to chitinases but lack chitinolytic activity [2], [3]. The murine AMCase shows sequence homology to Chit1, with an identity of 52% and a similarity of 60% [6]. The locus of the human Chit1 gene is found on chromosome 1q32 [7], whereas the human AMCase gene is located on chromosome 1p13 [6]. Both genes are composed of 12 exons and encode various splice isoforms [6]–[9]. The sequence homology and the conservation of the intron-exon boundaries between the Chit1 and AMCase genes suggest that these genes arose from a duplication of an ancestral gene [3], [6].

Recent studies have shown associations between the expression of the mammalian chitinases and inflammatory conditions. For instance, the levels of Chit1 are elevated in the plasma of patients with Gaucher disease, the bronchoalveolar lavage fluid of smokers and patients with chronic obstructive pulmonary disease (COPD) and the cerebrospinal fluid of patients with Alzheimer’s disease [9]–[12]. AMCase expression and activity are also upregulated during allergic airway responses in mouse models of asthma [13]. Polymeric chitin induces AMCase expression and the recruitment of immune cells that are associated with allergy and asthma [14]. In addition, several genetic variants of AMCase are associated with bronchial asthma in humans [15], [16]. These results strongly suggest that the chitinolytic enzymes play important roles in the response to disease. However, their pathophysiological functions remain poorly understood.

Quantification of Chit1 and AMCase mRNA levels is an important step toward the understanding of the *in vivo* regulation of chitinases in mammals. We recently established a quantitative PCR system using a single standard DNA to quantify and compare the expression levels of the chitinase and reference genes on the same scale [17]. In our previous paper, we showed that AMCase is a major transcript in the mouse stomach and is expressed at levels that are comparable to those of pepsinogen C [17].

Here, we applied our methodology to the quantification of mRNA levels of the mammalian chitinases in normal human tissues, which is a prerequisite for understanding their pathological roles in diseased tissues. Furthermore, we established a quantification system to compare the mRNA levels of multiple genes between a number of human and mouse tissues using a human-mouse hybrid standard DNA. Our study quantitatively shows that Chit1 mRNA is expressed at similarly high levels in normal human and mouse lungs. In contrast, AMCase is predominantly overexpressed in mouse but not human stomach.

## Results

### Establishment and Validation of a Real-time PCR System for Detection of Chitinases in Human Tissues

**Figure 1:**
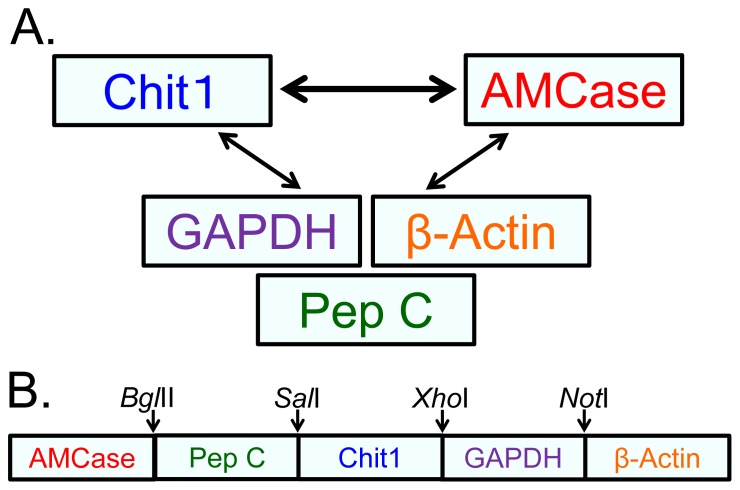
Strategy for the comparison of the gene expression levels of five human genes. (A) We compared the expression levels of the Chit1 and AMCase genes. To evaluate the chitinase levels, we used two housekeeping genes (GAPDH and β-actin) and pepsinogen C (progastricsin), which served as a reference gene for the stomach. Using these three reference genes, we evaluated the gene expression levels of Chit1 and AMCase in human tissues. (B) Schematic representation of the standard DNA used for real-time PCR. The target fragments for AMCase, pepsinogen C, Chit1, GAPDH and β-actin cDNAs, in addition to their flanking sequences and restriction sites, were ligated at a one-to-one ratio into a DNA fragment and used as the standard DNA for the analysis of the human genes.

We previously established a real-time PCR system that is capable of determining the mRNA levels of the two mammalian chitinases in mouse tissues and of comparing these levels with those of reference genes using the same scale [17]. In this study, we wanted to compare the gene expression levels of the Chit1 and AMCase genes across normal human tissues (Figure 1A). As in mouse tissues [17], we used the housekeeping genes glyceraldehyde-3-phosphate dehydrogenase (GAPDH) and β-actin as reference genes because they are constitutively expressed at high levels in most cells [18]. In addition, we chose pepsinogen C (also known as progastricsin) as a reference gene in the stomach because the level of AMCase mRNA in the mouse stomach was comparable to the mRNA level of pepsinogen C, which constitutes a major component of the gastric mucosa [19]. Using these three reference genes, we evaluated the gene expression levels of Chit1 and AMCase in normal human tissues (Figure 1A).

We designed several sets of primers for quantitative PCR and evaluated their suitability based on whether they exhibited a single melting temperature (Tm) and a single band on a 10% polyacrylamide gel [17]. As shown in **Figure S1**A–E, the dissociation curves of the five cDNAs exhibit only one peak each. Gel electrophoresis clearly showed single bands at the expected sizes for Chit1 (55 bp), AMCase (62 bp), GAPDH (57 bp), β-actin (57 bp) and pepsinogen C (61 bp) (**Figure S1**F). Thus, we confirmed that the correct PCR products were amplified from the human tissue cDNA mixture.

We next constructed a standard template DNA for real-time PCR by ligating the five target fragments in a one-to-one ratio (Figure 1B and **Figure S2**). The 1,396-nucleotide-long standard template DNA consisted of five cDNA fragments that covered the PCR target region and 60–143 bases of the flanking regions and contained *Bgl*II, *Sal*I, *Xho*I and *Not*I restriction sites (Figure 1B and **Figure S2**).

The quantification of both the chitinases and the reference mRNAs relies on standard curves. We used the standard curves to compare and evaluate the real-time PCR quantification strategies. Each standard curve was generated using 10-fold serial dilutions of the human standard DNA and the five different primer pairs (**Figure S3**A–E, red closed circles). By using the standard template DNA containing the five human cDNA fragments, equal quantities can be assigned to all five genes in each dilution use to construct the standard curves (**Figure S3**A–E, red closed circles).

To test the equality of the curves, a known concentration of the human entire coding cDNA was amplified and subsequently analyzed as an unknown sample. This assay was performed to verify that each tested dilution resulted in the expected quantity. As shown in **Figure S3**A–E, blue closed rhombuses, equal quantities were observed for each tested dilution; these were used to construct the standard curve.

The quantification of low-abundance transcripts and abundant transcripts allows us to validate the sensitivity and reliability of real-time PCR, indicating that our real-time PCR method offers a large dynamic range of quantification, high accuracy, and high sensitivity (**Figure S3**A–E). Thus, our real-time PCR method provides reliable values for the two human chitinase genes and the three human reference genes on the same scale.

### Expression of Chit1 and AMCase in Normal Human Tissues

**Figure 2:**
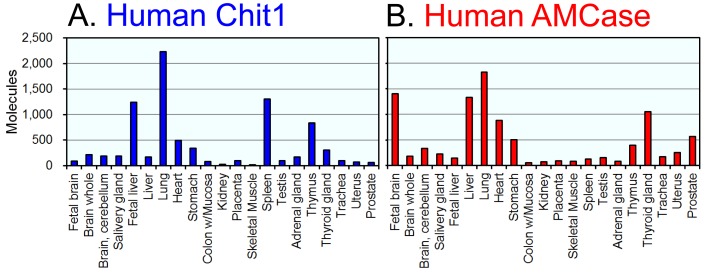
Expression of Chit1 and AMCase mRNAs in normal human tissues. Quantification of Chit1 (A) and AMCase (B) mRNAs in human tissues. Both chitinases were quantified by real-time PCR using the human standard DNA. All of the values are expressed as number of molecules per 10 ng of total RNA in y axis.

**Figure 3:**
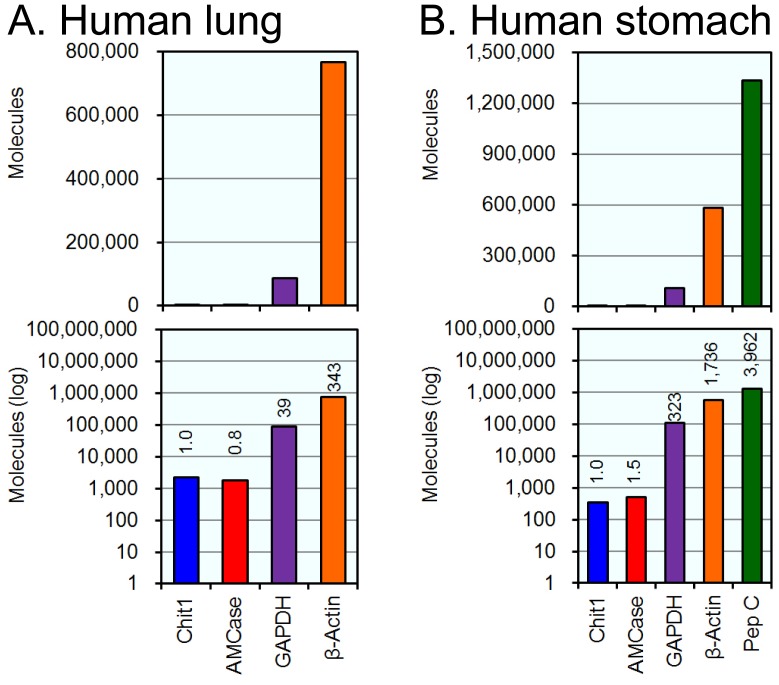
Analysis of Chit1 and AMCase mRNAs and reference gene mRNAs in lung and stomach tissues. The expression levels of the five genes, which were determined using cDNAs prepared from normal human lung (A) and stomach (B) tissues, were quantified by real-time PCR. The upper panel indicates the actual value, and the lower panel shows the logarithm of each value. The expression level of the human Chit1 gene was set to 1.0; the values on the bars indicate the relative expression levels compared to the expression level of the human Chit1 gene.

To study the *in vivo* regulation of the expression of the human Chit1 and AMCase genes, total RNA from various normal human tissues was analyzed using a quantitative real-time PCR assay with the specifically designed standard DNA (Figure 1). The resulting values were expressed as molecules per 10 ng of total RNA in y axis (Figure 2 and Figure 3).

Both Chit1 and AMCase mRNAs were widely expressed in normal human tissues (Figure 2A and 2B). The highest levels of Chit1 mRNA were detected in human lung, followed by spleen, fetal liver and thymus (Figure 2A). The highest levels of AMCase mRNA were detected in lung, followed by fetal brain, liver, thyroid gland and heart (Figure 2B). Although AMCase mRNA was expressed at extraordinarily high levels in the mouse stomach [17], its expression level in the human stomach was much lower than those in lung, fetal brain, fetal liver, thyroid gland and heart (Figure 2B). In other tissues, both the Chit1 and AMCase mRNAs were expressed at low, but easily detectable levels above background (Figure 2A and Figure 2B).

When compared with AMCase mRNA levels, Chit1 mRNA was expressed at relatively higher levels in spleen and fetal liver. In contrast, fetal brain, prostate and liver expressed a higher amount of AMCase mRNA than Chit1 mRNA (Figure 2).

### Analysis of the Expression Levels of Chit1, AMCase, GAPDH, β-actin and Pepsinogen C mRNAs in Normal Human Lung and Stomach Tissues

Because many studies on the pathophysiology of mammalian chitinases have been performed using lung and stomach [6], [8], [9], [13], [14], [20]–[23], we compared the expression levels of the chitinases and the reference genes in these two human tissues. The quantitative data are shown in Figure 3. Normalizing the Chit1 level to 1.0, the relative expression levels of AMCase, GADPH, and β-actin mRNAs in human lung tissue were 0.8, 39 and 343, respectively (Figure 3A). The GAPDH and β-actin genes are well-known housekeeping genes and are constitutively expressed at high levels in most tissues [18]. Our results indicate that Chit1 and AMCase mRNAs are expressed at similar levels in normal human lung tissue, although the Chit1 expression level was much lower than those of the two housekeeping genes (compare non-log plot with log plot in Figure 3).

Normalizing the Chit1 level to 1.0, the relative expression levels of the AMCase, GAPDH, β-actin and pepsinogen C in normal human stomach tissue were 1.5, 323, 1,736 and 3,962, respectively (Figure 3B). Here, the expression levels of both Chit1 and AMCase were much lower than those of GAPDH and β-actin. Although pepsinogen C mRNA was highly expressed in the human stomach samples, AMCase mRNA was comparable to Chit1. These results indicate that the Chit1 and AMCase expression levels are relatively low in the human tissues examined and that the AMCase expression level in the stomach differs significantly between human and mouse.

### Establishment and Validation of a Quantification System for Human and Mouse Chitinase mRNAs Using a Human-mouse Hybrid DNA

**Figure 4:**
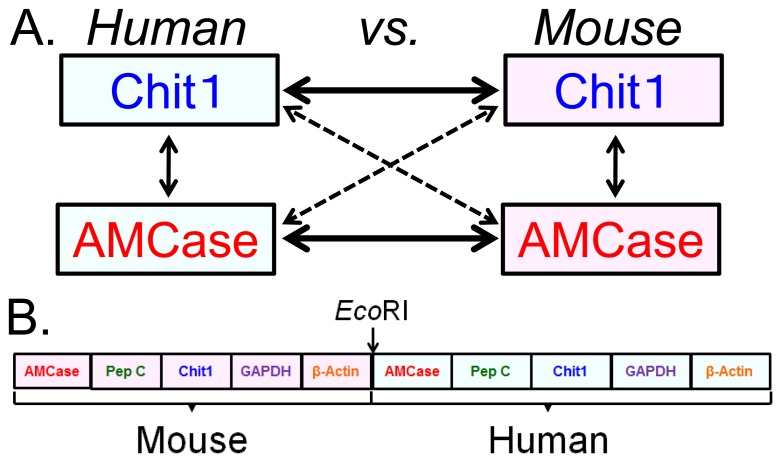
Strategy for comparing Chit1 and AMCase mRNA levels between human and mouse tissues. (A) The expression levels of the Chit1 and AMCase genes in human and mouse tissues were compared. (B) Schematic representation of the human-mouse hybrid standard DNA used for the quantification. The human and mouse standard DNAs were ligated using the *Eco*RI site at a one-to-one ratio into a DNA fragment and used as the human-mouse hybrid standard DNA.

We next sought to compare the expression levels of the two chitinases between humans and mice on the same scale using a real-time PCR system (Figure 4A). We therefore constructed a human-mouse hybrid standard DNA for real-time PCR by ligating the human and mouse standard template DNAs (Figure 4B). The resulting 2,305-nucleotide-long template DNA contained ten cDNA fragments that covered the PCR target region and 9–143 bases of the flanking regions of the human and mouse genes (see details in **Figure S4**).

The validations of the standard curve and the quantitative real-time PCR system were performed as shown in **Figure S5**, **Figure S6** and **Figure S7**. Serial dilutions of the human-mouse hybrid standard DNA were used to construct a standard curve. Each standard curve was generated using 10-fold serial dilutions of the standard DNA and the five different primer pairs (**Figure S5**A–E, red closed circles). By using the standard template DNA containing the five cDNA fragments, equal quantities can be assigned to all of the five human genes using human-specific primer pairs in each dilution that was used to construct the standard curves (**Figure S5**A–E, red closed circles). Similarly, equal quantities can be assigned to all of the five murine genes (**Figure S**6A–E, purple closed triangles).

To test the equality of the curves, a known concentration of the entire coding cDNA was amplified and subsequently analyzed as an unknown sample. Equal quantities of the human entire coding cDNAs were observed for each tested dilution; these quantities were then used to construct the standard curve (**Figure S5**A–E, blue closed rhombuses). Similarly, equal quantities of the mouse entire coding cDNAs were also observed (**Figure S6**A–E, green crosses). The quantification of low-abundance transcripts and abundant transcripts allows us to validate the sensitivity and reliability of real-time PCR, indicating that our real-time PCR method offers a large dynamic range of quantification, high accuracy, and high sensitivity (**Figure S5**A–E and **Figure** 6A–E).

Furthermore, the four lines (two standard curves and two dilution curves of the known concentrations of the human and mouse entire coding cDNAs, respectively) overlapped (**Figure S7**A–E). Thus, our real-time PCR method provides reliable relative values for the four chitinase genes and the six reference genes (Figure 4B).

### Comparison of Chit1 and AMCase mRNA Levels between Normal Human and Mouse Tissues

**Figure 5:**
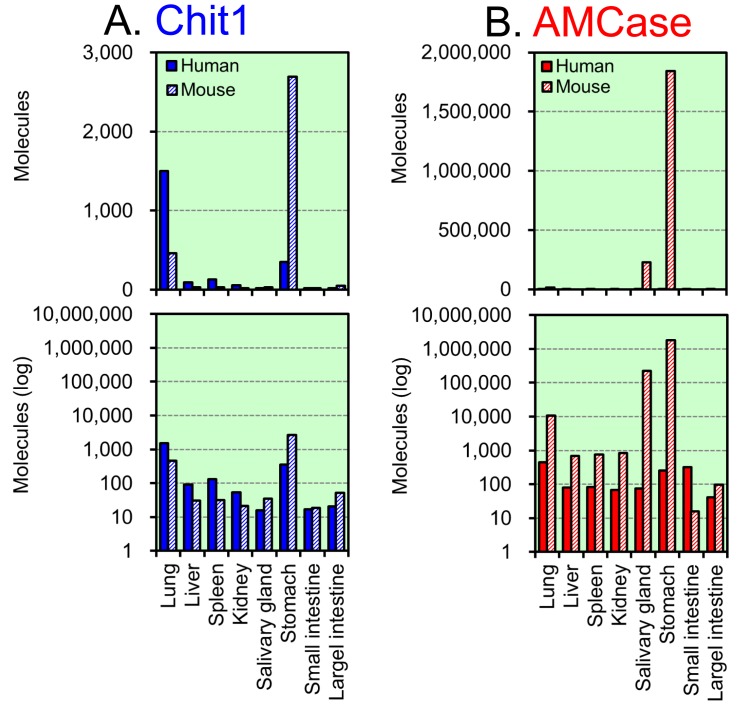
Expression of Chit1 and AMCase mRNAs in human and mouse tissues. The expression levels of Chit1 (A) and AMCase (B) mRNAs in eight human and mouse tissues were quantified on the same scale by real-time PCR using the hybrid standard DNA. Filled bars, human tissues; hatched bars, mouse tissues. All of the values are expressed as number of molecules per 10 ng of total RNA in y axis. The upper panel indicates the actual value, and the lower panel shows the logarithm of each value. Part of mouse data has been reported in our previous paper [17].

We used the Human Total RNA Master Panel II and the Mouse Total RNA Master Panel (Clontech Laboratories) as sources of total RNA for this study. There are four tissues (lung, liver, spleen and kidney) that overlapped between the human and mouse panels and related to asthma and Gaucher disease. Since there were prominent differences in the chitinase expression in the stomach tissues, we also looked at the levels of expression of these chitinases in other digestive organs, salivary gland, stomach, small and large intestines between human and mouse. We quantified and compared the expression levels in these tissues using the human-mouse hybrid standard DNA (Figure 4B); the quantitative data are shown in Figure 5.

**Figure 6:**
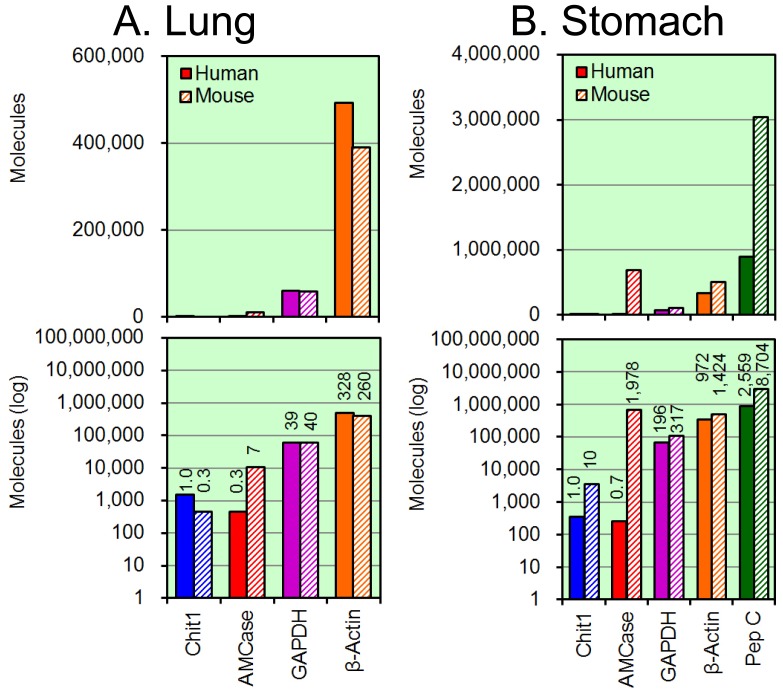
Analysis of the expression levels in human and mouse lung and stomach tissues. The expression levels of the five genes, which were determined using the cDNAs prepared from normal human and mouse lung (A) and stomach (B) tissues, were quantified by real-time PCR. Filled bars, human tissues; hatched bars, mouse tissues. The upper panel indicates the actual value, and the lower panel shows the logarithm of each value. The expression level of the human Chit1 gene was set to 1.0; the values on the bars indicate the relative expression levels compared to the expression level of the human Chit1 gene. Part of mouse data has been reported in our previous paper [17].

We found the highest expression level of Chit1 mRNA in mouse (but not human) stomach, followed by human lung tissues. Overall, Chit 1 mRNA was expressed at higher levels in the human tissues than in the mouse tissues except for stomach (Figure 5A). The highest expression level of AMCase mRNA was in mouse stomach, followed by mouse salivary gland. In the other human and mouse tissues, the AMCase mRNA levels were low in human tissues (Figure 5B). Both Chit1 and AMCase mRNA levels in small and large intestines were very low in both human and mouse tissues (Figure 5), which were consistent with previous data obtained by Northern blotting [6], [21].

We found that AMCase mRNA was expressed at low levels in normal human stomach but was highly expressed in mouse stomach tissues (Figure 5B). Thus, we also compared the expression levels of the chitinases and the reference genes using the cDNAs that were prepared from the lung and stomach tissues.

Normalizing the human Chit1 level in the lung tissue to 1.0, the relative expression levels of the mouse Chit1, human AMCase and mouse AMCase mRNAs were 0.3, 0.3 and 7, respectively (Figure 6A). Murine AMCase was highly expressed in the mouse lung. The level of Chit1 mRNA in the human lung was about 3-fold higher than that in mouse lung. The Chit1 and AMCase mRNA levels were significantly lower than the expression levels of the GAPDH and β-actin genes (Figure 6A).

Normalizing the human Chit1 level in stomach to1.0, the relative expression levels of the mouse Chit1, the human AMCase and the mouse AMCase mRNAs were 10, 0.7 and 1,978 respectively (Figure 6B). Pepsinogen C is a major digestive enzyme in the stomach. The expression level of AMCase in mice was much higher than those of GAPDH and β-actin and was comparable to the level of pepsinogen C, whereas the human stomach exhibited a very low level of AMCase mRNA expression. The relative expression levels of mRNA in the human and mouse stomach were 1.0 and 2,826, respectively.

**Figure 7:**
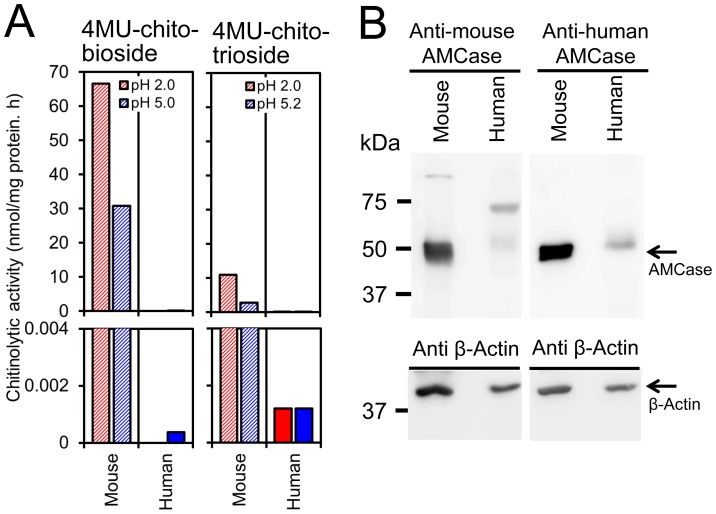
The levels of chitinases activity and protein expression in mouse and human stomach tissues. (A) The chitinolytic activity in the stomach extracts from mouse and human stomach tissues. Filled bars, human tissues; hatched bars, mouse tissues. (left) AMCase activity was determined using the synthetic chitin substrate of 4MU-chitobioside. Chitinolytic activity at pH 2.0 and pH 5.0 were expressed in y axis. (right) Chit1 activity was measured using 4MU-chitotriose at pH 5.2, as previously described in Materials and Methods. AMCase activity was also measured at pH 2.0. All graph points are the mean of triplicate measurements and representative of multiple experiments. (B) Representative patterns of Western blotting of mouse and human AMCase in mouse or human stomach proteins. Proteins were run on SDS-polyacrylamide gels and analyzed by Western blotting using anti-human or anti-mouse AMCase antibodies. Stomach soluble proteins (6.3 µg) were separated on SDS-polyacrylamide gels and transferred onto Immobilon membranes, and probed with AMCase specific antibodies.

In order to check whether the mRNA level differences between mouse and human were reflected at the protein level, we next analyzed enzymatic activity of these chitinases in the stomach tissues. We first analyzed AMCase activity in mouse and human stomach tissues. The mouse AMCase shows a dual pH optimum with a major optimum around pH 2 and a secondary optimum around pH 5 [6], whereas human AMCase shows broad optimal pH at pH 2∼pH 5 [16], [24]. Thus, we measured chitinolytic activity at pH 2.0 and pH 5.0 using the synthetic substrate of 4-methylumbelliferyl β-D-N, N′-diacetylchitobiose (4MU-chitobiose) [6], [21]. We detected robust chitinolytic activity in mouse stomach extract at pH 2.0 and strong activity at pH 5.0. In contrast, no activity was detected in that of human at pH 2.0 and very low activity was observed at pH 5.0 (Figure 7A, left).

The standard Chit1 enzymatic assay has been performed using 4-methylumbelliferyl β-D-N, N′, N′′-triacetylchitotriose (4MU-chitotriose) at pH 5.2 [6], [10], [21], [25]. To monitor the effect of AMCase on Chit1 activity, we analyzed the chitinase activity using 4MU-chitotriose at pH 2.0. Relatively strong chitinase activity against 4MU-chitotriose was detected in the mouse stomach extract at pH 2.0 (Figure 7A right), and we also detected weak chitinase activity at pH 5.2 (Figure 7A right). Since AMCase has a second optimum at around pH 5, these results indicated that most of chitinolytic activity at pH 5.2 may be due to AMCase rather than Chit1 and that mouse AMCase could hydrolyze 4MU-chitotriose as substrate at pH 2.0. Taken together, the majority of chitinase activity in the mouse stomach resulted from AMCase activity. In human stomach extract we also observed very weak but detectable levels of chitinolytic activity at pH 2.0 and pH 5.2 (Figure 7A, right, lower panel). Chitinase activity at pH 5.2 was comparable to that at pH 2.0, indicating Chit1 expression in human stomach.

Finally, we analyzed the levels of protein expression by Western blotting using antibodies against AMCase. The anti-AMCase antibodies were generated against the previously reported mouse peptide [14] and human counterpart. Anti-mouse AMCase antibody recognized a robust single protein band in extract from mouse stomach but not from human (Figure 7B, left). Similarly anti-human AMCase antibody also recognized a single band in the mouse extract (Figure 7B, right). In the human extract there were faint bands of slightly higher molecular weight that were recognized by both human and mouse antibodies (Figure 7B). Since there was no significant chitinase activity at either pH 2.0 or pH 5.0 monitored by using 4MU-chitobiose in human stomach extract, these bands could not be related to the human AMCase. Consequently, chitinolytic activities and the relative protein expression levels between mouse and human stomach tissues were consistent with our data obtained at the mRNA level.

## Discussion

Previous studies on the *in vivo* regulation of Chit1 and AMCase in human and mouse were carried out using northern blotting, semi-quantitative PCR and real-time PCR [6], [8], [9], [13], [14], [22], [23]. Although these methods have several advantages over the examination of gene expression patterns, they failed to compare the levels of the different gene transcripts on the same scale. In the current study, we applied our methodology [17] to investigate the expression levels of these chitinases in human tissues. Furthermore, to evaluate the expression levels of chitinases in human and mouse tissues, we developed a quantification system using a single human and mouse hybrid standard DNA. We showed tissue- and species-specific expression of the two mammalian active chitinases, Chit1 and AMCase. Our data generally supports previous studies reported by Boot et al [6], [21]. However, our analysis is sufficiently sensitive to detect mRNA and provides a comprehensive survey of the gene expression patterns of the chitinases in human and mouse tissues on the same scale.

One noticeable characteristic of the expression of the mammalian chitinases is the conserved expression of Chit1 between human and mouse lung tissues. We found that Chit1 was expressed at relatively high levels in human and mouse lung tissues that were nearly comparable with each other, indicating that the expression level of the Chit1 mRNA is conserved. In the lungs, Chit1 can act as part of the host defense system that protects against chitin-containing pathogens, such as fungi and mites [25]. The conservation of Chit1 gene expression in humans and mice suggests the physiological importance of Chit1 in the lung.

The expression of chitinases in the stomach is of particular interest. AMCase mRNA is synthesized at extraordinarily high levels in the mouse stomach but not in the human stomach (Figure 6B). The comparison of these expression levels showed that the AMCase mRNA level in the human stomach was about 1/2,800 of that in the mouse counterpart. We confirmed that the expression levels of pepsinogen C mRNA and the two housekeeping genes were very high in the human and mouse stomach tissues (Figure 6B). Thus, the decreased expression level of AMCase mRNA in the human stomach is not due to RNA degradation during the cDNA preparation. Furthermore, we found that mouse stomach expressed large amount of AMCase, whereas the human counterpart did not (Figure 7). These results indicate that the expression level of the AMCase in the stomach tissues differs significantly between humans and mice.

The stomach is an important organ that plays fundamental roles in the digestion of foods and protection against harmful organisms. Hydrochloric acid is secreted in the stomach, creating acidic conditions (pH = ∼2) appropriate for the digestion of proteins by pepsin [19], [26]. Murine AMCase shows profound acid stability and is most active at around pH 2 [6]. The mouse stomach produces an enormous amount of AMCase mRNA [17] and its translation product (Figure 7). Consequently, AMCase can function as a digestive enzyme that breaks down chitin-containing foods in the mouse stomach.

In contrast, the expression level of AMCase mRNA and its product were relatively low in the human stomach (Figure 5, Figure 6 and Figure 7). This is not unexpected because modern humans do not eat a significant amount of chitin-containing foods. These results suggest that AMCase may not play a role in the defense against chitin-containing organisms in the human stomach. Many stomach diseases are associated with infection by exogenous organisms. The severity of gastritis-associated infections such as *Helicobacter pylori* may correlate with the activity of endogenous enzymes [23]. It thus remains a matter of debate whether the low level of AMCase in the human stomach participates in the response to gastric disorders.

A high level of chitinolytic activities have been detected in extracts of mouse stomach and intestine [6]. Since there are prominent differences in the levels of chitinase expression in the stomach between human and mouse, we examined the levels of expression of these chitinases in other digestive organs, including salivary gland and small and large intestines. Our results indicate that both Chit1 and AMCase mRNA levels in small and large intestines were very low in both human and mouse tissues, although mouse salivary gland and stomach produced high levels of both chitinases mRNAs (Figure 5). These results suggest that mammalian chitinase proteins in the mouse intestines are probably derived from the upper parts of the gastrointestinal tract, such as salivary gland and stomach, which is consistent with the prior notion by Boot et al [6], [21].

The expression levels of the Chit1 gene in lung tissues is conserved between humans and mice. In contrast, the expression level of the AMCase in stomach tissues is species-specific. The results suggest that the regulation of the expression of Chiti1 mRNA in the lung has been preserved during evolution, whereas the decreased expression of AMCase in the human stomach might be the result of gene silencing. The examination of the regulation of the expression of these mammalian chitinases could give insights into the pathophysiological roles of these enzymes. A detailed characterization of the promoter regions of the Chit1 and AMCase genes and the identification of the *cis*- and *trans*-acting factors will be required for the understanding of the selective gene expression of these chitinases in humans and mice.

The expression of Chit1 and AMCase mRNAs is induced under various pathological conditions. Using the quantitative system described here, we will be able to compare the chitinase mRNA levels across human and mouse tissues by real-time PCR. This analysis will aid the understanding of the biological function of these chitinases, especially in the pathophysiologic studies of human disease using murine models. Our methodology is applicable to the quantification of the mRNAs of multiple genes between human and mouse specimens using the same scale. A practical use of this comparative cross-species gene expression data is the comparison of human diseases with mouse and cell culture models.

## Materials and Methods

### RNA and cDNA Preparation

The Human Total RNA Master Panel II and the Mouse Total RNA Master Panel (Clontech Laboratories) were used to examine the distribution of transcripts in various tissues. Human Small Intestine Total RNA was purchased from Clontech Laboratories. In addition, RNA was isolated from salivary gland and small and large intestines of 3-month-old male mice. C57BL/6J mice (CLEAR Japan) were bred at the RIKEN Brain Science Institute Animal Facility. All animal experiments were performed in compliance with the institutional guidelines. The protocol was approved by the Committee on the Ethics of Animal Experiments of the RIKEN Brain Science Institute (Approval No. H19-2B013). All surgery was performed using diethyl ether as an anesthetic, and all efforts were made to minimize suffering. Tissues for mRNA preparation were provided by Drs. Miyazaki and Nukina at RIKEN Brain Science Institute. Total RNA was prepared from salivary gland and small and large intestines using TRIzol Reagent (Invitrogen) according to the manufacturer’s instructions. We analyzed 21 different human tissues and eight adult mouse tissues. To remove trace amounts of contaminating genomic DNA, total RNA samples were treated with RQ1 RNase-free DNase (Promega) according to the manufacturer’s recommendations. Each of the total RNA samples was subjected to reverse transcription using random hexamers as the primers, as reported previously [17].

### Real-time PCR

The establishment and validation of the real-time PCR system for human tissues were performed as described [17]; the only exception was the use of human primers. The nucleotide sequences of the primers that were selected for the real-time PCR for the human system are shown in **Table S1**. Each sample was amplified in triplicate, and each experiment was repeated at least two times.

### Construction of the Human Standard DNA and Preparation of Five Human cDNAs Covering the Entire Coding Region

The construction of the standard template DNA for the human genes and the preparation of the five human cDNAs that covered the entire coding region were performed essentially as described [17]; the only exception was the use of human primers. The forward and reverse primers are listed in **Table S2**. The PCR products were digested with the appropriate restriction enzymes and ligated using T4 DNA ligase. The ligated fragments were amplified using the forward primer 5′-CATGGAATTCTGGTCTGGGCCATTGATCTGGATG-3′ and the reverse primer 5′-CAATCTCATCTTGTTTTCTGCGCAAGTTAGG-3′ and cloned into the pGEM-T Easy vector (Promega). The verified sequences of the cDNAs are shown in **Figure S2**. The standard DNA (1,396 bases; see Figure 1B and **Figure S2**) was prepared by PCR reamplification from the plasmid DNA using the same primers and was thereafter used as the standard DNA.

The human entire coding cDNAs were amplified by PCR (using the primer sets listed in **Table S3**) and subcloned into the pGEM-T Easy vector. The sequences of the cDNAs were verified by sequencing (**Figure S8)**. The subcloned fragments were reamplified from the plasmid DNAs using the same primers and used as the entire coding region cDNAs.

### Standard Curves and Quantification of mRNAs by Real-time PCR

The molar concentration of the multigene-containing human standard DNA was calculated based on the concentration and the molecular weight. The standard curves were constructed and the mRNA quantification performed as described [17].

### Construction of the Human-mouse Hybrid Standard DNA

The standard DNA (2,305 bases; see Figure 4B and **Figure S4**) used for the quantification of the transcript levels by real-time PCR was constructed as follows. The mouse standard DNA, which contained the *Eco*RI restriction site at the 3′ end, was amplified by PCR using the forward primer 5′-GTGGATTCTGTGCCGACAAAGCAGATGGCC-3′ and the reverse primer 5′-CATGGAATTCTGGGTACATGGTGGTACCACCAGA-3′. The human standard DNA with *Eco*RI restriction site at the 5′ end (see **Figure S2**) was prepared as described above. The PCR products were digested with *Eco*RI, purified using agarose gel electrophoresis and then ligated using T4 DNA ligase. The ligated fragments were amplified by PCR using the forward primer 5′-GTGGATTCTGTGCCGACAAAGCAGATGGCC-3′ and the reverse primer 5′-CAATCTCATCTTGTTTTCTGCGCAAGTTAGG-3′. The resulting DNA was cloned into the pGEM-T Easy vector. The verified sequences of the cDNA are shown in **Figure S4**. The linearized human-mouse standard DNA was prepared by PCR reamplification from the plasmid DNA using the same primers and used as the standard DNA. The standard curves were constructed and the mRNA quantification performed as described above.

### Antibody Preparation

Rabbit polyclonal antibodies specific to mouse and human AMCase were produced by Sigma-Aldrich Life Science Japan. Cys-peptides were conjugated through the added C-terminal cysteine to keyhole limpet hemocyanin (KLH). Sera from immunized rabbits were affinity-purified by use of the antigen with Cys (i.e. mouse AMCase, KADGLYPVADDRNAFWQC; human AMCase, RANGLYPVANNRNAFWHC) coupled to Sulfolink (Pierce). The specificity of each antibody was confirmed by immunoblot assays.

### Mouse Stomach Protein Preparation and Human Stomach Lysate

Stomach tissue isolated from 3-month old C57Bl/6J mice was homogenized in 10 volumes of ice-cold 20 mM Tris-HCl buffer (pH 7.6), containing 150 mM NaCl and protease inhibitors (Complete Protease Inhibitor Cocktail Tablet, Roche Diagnostics) using a Teflon/glass homogenizer. The homogenates were centrifuged at 17,000 g for 10 min at 4°C, and the supernatants were kept. Total protein extract of normal human stomach tissue was obtained commercially [Cat. no. NB820-59263; Whole Normal Fundus Stomach Tissue Lysate (Adult Normal), Novus Biologicals]. Protein concentrations in each fraction were determined by the Protein Assay (Bio-Rad).

### Chitinase Enzymatic Assays

Chitinase enzyme activity was determined with the fluorogenic substrate 4MU-chitobiose or 4MU-chitotriose using Chitinase Assay Kit, Fluorimetric (Sigma-Aldrich) according to the manufacturer’s instructions and as described previously [4], [6], [10], [21]. The fluorescence of liberated 4MU was measured using a fluorometer (RF-5300PC, Shimazu) with excitation at 360 nm and emission at 450 nm. The amount of product (4MU) was estimated using a standard curve based on 4MU (Sigma-Aldrich).

### Immunoblotting

We loaded 6.3 µg of stomach protein on a SDS-polyacrylamide gel (10% gel) [27] and separated proteins were transferred electrophoretically onto Immobilon (Millipore), which was probed with anti-human or anti-mouse AMCase antibody, followed by peroxidase-conjugated AffiniPure F (ab′)_2_ Fragment Donkey Anti-Rabbit IgG (H+L) (Jackson ImmunoResearch laboratories). Bound antibodies were detected using Immobilon Western Chemiluminescent HRP Substrate (Millipore). For confirmation of the loading control, the membrane was re-probed with monoclonal anti-β-actin (clone AC-15) (Sigma-Aldrich), followed by anti-mouse IgG (H+L) (Jackson ImmunoResearch laboratories). Signals were detected using a Luminescent Image Analyzer (ImageQuant LAS 4000, GE Healthcare).

## Supporting Information

Figure S1 **Evaluation of the primer pairs that are suitable for the human real-time PCR system.** The PCR primers for the human analysis were selected based on whether they exhibited one melting temperature (A–E) and a single PCR product on a 10% polyacrylamide gel (F). The dissociation curves of the PCR products of the five genes were generated using a human tissue cDNA mixture. The PCR products were analyzed on a 10% polyacrylamide gel stained with ethidium bromide.
(TIF)

Figure S2 **Nucleotide sequence of the human standard DNA.** The human standard DNA (1,396 nucleotides long) contained *Eco*RI restriction site at the 5′ end (shown in plain text) and five cDNA fragments (shown in different colors) that covered the PCR target regions (shown in bold and underlined) and 60–143 bases of the flanking regions and contained the *Bgl*II, *Sal*I, *Xho*I and *Not*I restriction sites (shown in bold and italics).
(TIF)

Figure S3 **Development and validation of a quantitative real-time PCR system for the analysis of human tissues.** The analyzed cDNAs were the following: A, Chit1; B, AMCase; C, GAPDH; D, β-actin; and E, pepsinogen C. Standard curves were obtained using the standard DNA containing the five human cDNA fragments (red closed circles). In addition, the quantification of the human entire coding cDNA was performed using the primer pairs for each gene. The target cDNA was amplified from a dilution of the entire coding cDNA with a known concentration and subsequently analyzed as an unknown sample (blue closed rhombuses). Equal quantities were obtained for each tested dilution of the standard curve and entire coding cDNA.
(TIF)

Figure S4 **Nucleotide sequence of the human-mouse hybrid standard DNA.** The human and mouse standard DNAs were ligated using the *Eco*RI site (shown in larger font, bold and italics) at a one-to-one ratio into a DNA fragment that was subsequently used as the human-mouse hybrid standard DNA. The 2,305-nucleotide-long DNA contained ten cDNA fragments (shown in different colors) that covered the PCR target regions (shown in bold and underlined) and 9–143 bases of the flanking regions of the human and mouse genes; it contained the appropriate restriction sites (shown in bold and italics).
(TIF)

Figure S5 **Development and validation of a real-time PCR system using the human-mouse hybrid standard DNA for the analysis of human genes.** The analyzed human DNAs were the following: A, Chit1; B, AMCase; C, GAPDH; D, β-actin; and E, pepsinogen C. The standard curves were obtained using the hybrid standard DNA containing the five human cDNA fragments (red closed circles). In addition, the quantification of the human entire coding cDNAs was performed using primer pairs for each gene. The target cDNA was amplified from a dilution of the entire coding cDNA with a known concentration (see **Figure S8**) and subsequently analyzed as an unknown sample (blue closed rhombuses). Equal quantities were observed for each tested dilution of the standard curve and entire coding cDNA.
(TIF)

Figure S6 **Development and validation of a real-time PCR system using the human-mouse hybrid standard DNA for the analysis of mouse genes.** The same experiments as those shown in **Figure S5** were performed for the mouse genes. The standard curves were obtained using the hybrid standard DNA containing the five mouse cDNA fragments (purple closed triangles). In addition, the quantification of the mouse entire coding cDNAs was performed using primer pairs for each gene. The target cDNA was amplified from a dilution of the entire coding cDNA with a known concentration and subsequently analyzed as an unknown sample (green crosses). Equal quantities were observed for each tested dilution of the standard curve and entire coding cDNA.
(TIF)

Figure S7 **Development and validation of a quantitative real-time PCR system using the standard DNA.** The results in **Figure S5** and **Figure S6** were superimposed. The quantification of low-abundance and abundant human and mouse transcripts allowed validation of the sensitivity and reliability of the real-time PCR system. The results indicate that our real-time PCR system and the human-mouse hybrid standard DNA offer a large dynamic quantification range that exhibits high accuracy and high sensitivity.
(TIF)

Figure S8 **Nucleotide sequence and calculated molecular weight of the human entire coding cDNAs.**
(DOC)

Table S1 **The nucleotide sequences of the primers that were selected for the real-time PCR for the human system.**
(DOC)

Table S2 **Forward and reverse primers used to construct the human standard template DNA for real-time PCR.**
(DOC)

Table S3 **Primers for PCR amplification of the human entire coding cDNAs.**
(DOC)
