# Supplementary figures and images for: Quantification of Chitinase mRNA Levels in Human and Mouse Tissues by Real-Time PCR: Species-Specific Expression of Acidic Mammalian Chitinase in Stomach Tissues
Source: PLoS One. 2013 Jun 27;8(6):e67399. doi: 10.1371/journal.pone.0067399 (PMC3694897; doi:10.1371/journal.pone.0067399)

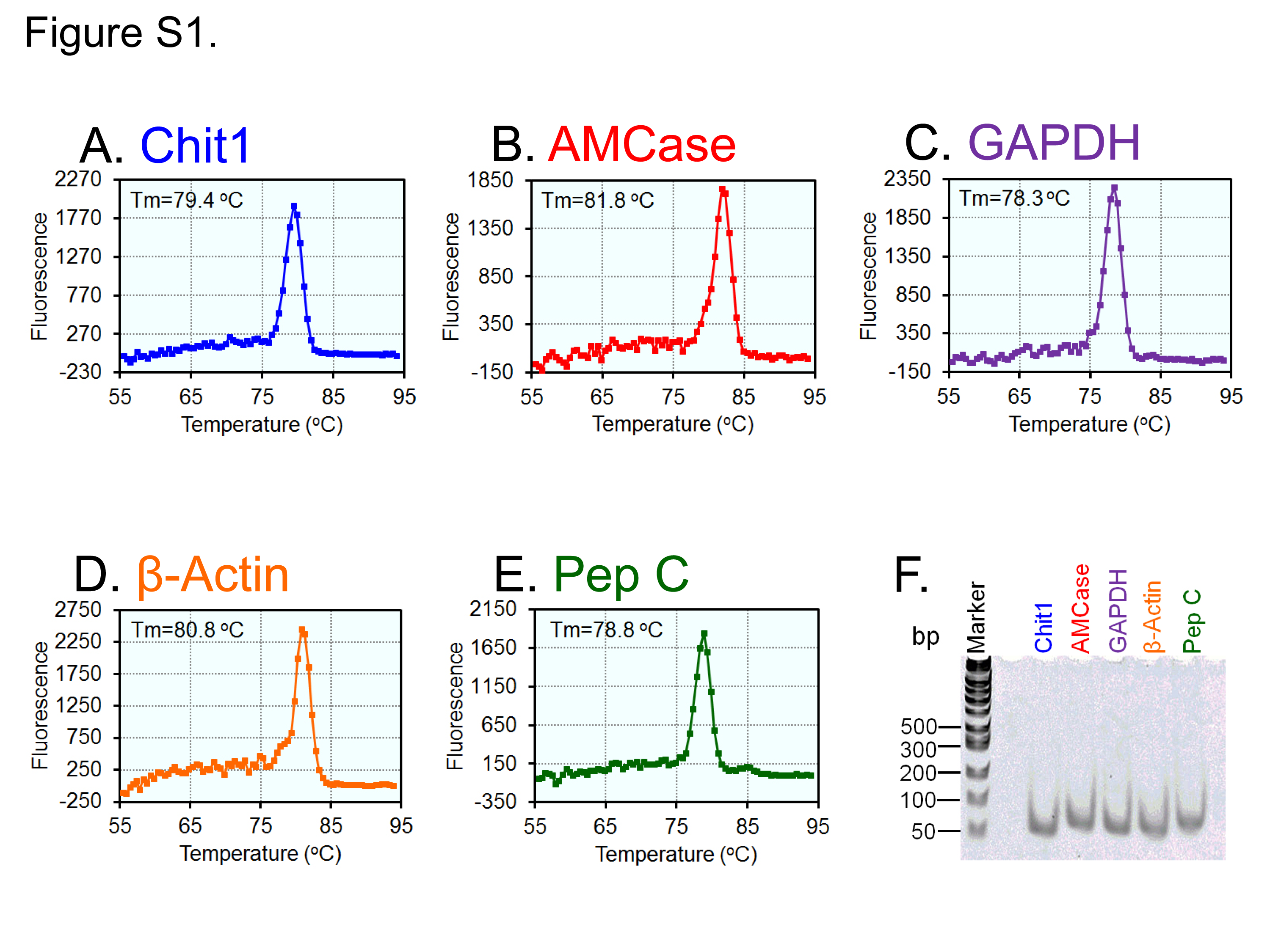

Supplement: Figure S1 — Evaluation of the primer pairs that are suitable for the human real-time PCR system. The PCR primers for the human analysis were selected based on whether they exhibited one melting temperature (A–E) and a single PCR product on a 10% polyacrylamide gel (F). The dissociation curves of the PCR products of the five genes were generated using a human tissue cDNA mixture. The PCR products were analyzed on a 10% polyacrylamide gel stained with ethidium bromide. (TIF) [file pone.0067399.s001.tif]

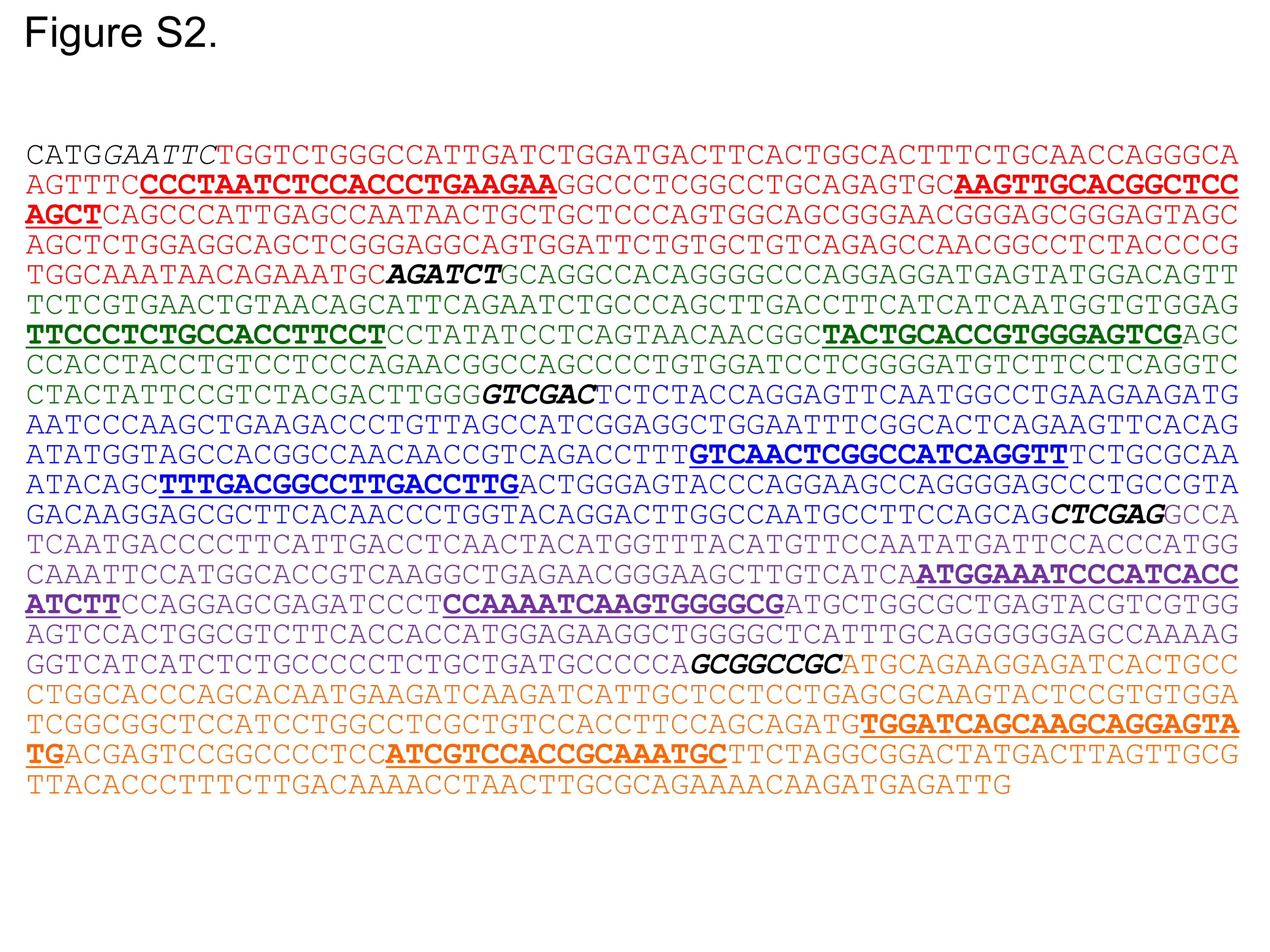

Supplement: Figure S2 — Nucleotide sequence of the human standard DNA. The human standard DNA (1,396 nucleotides long) contained EcoRI restriction site at the 5′ end (shown in plain text) and five cDNA fragments (shown in different colors) that covered the PCR target regions (shown in bold and underlined) and 60–143 bases of the flanking regions and contained the BglII, SalI, XhoI and NotI restriction sites (shown in bold and italics). (TIF) [file pone.0067399.s002.tif]

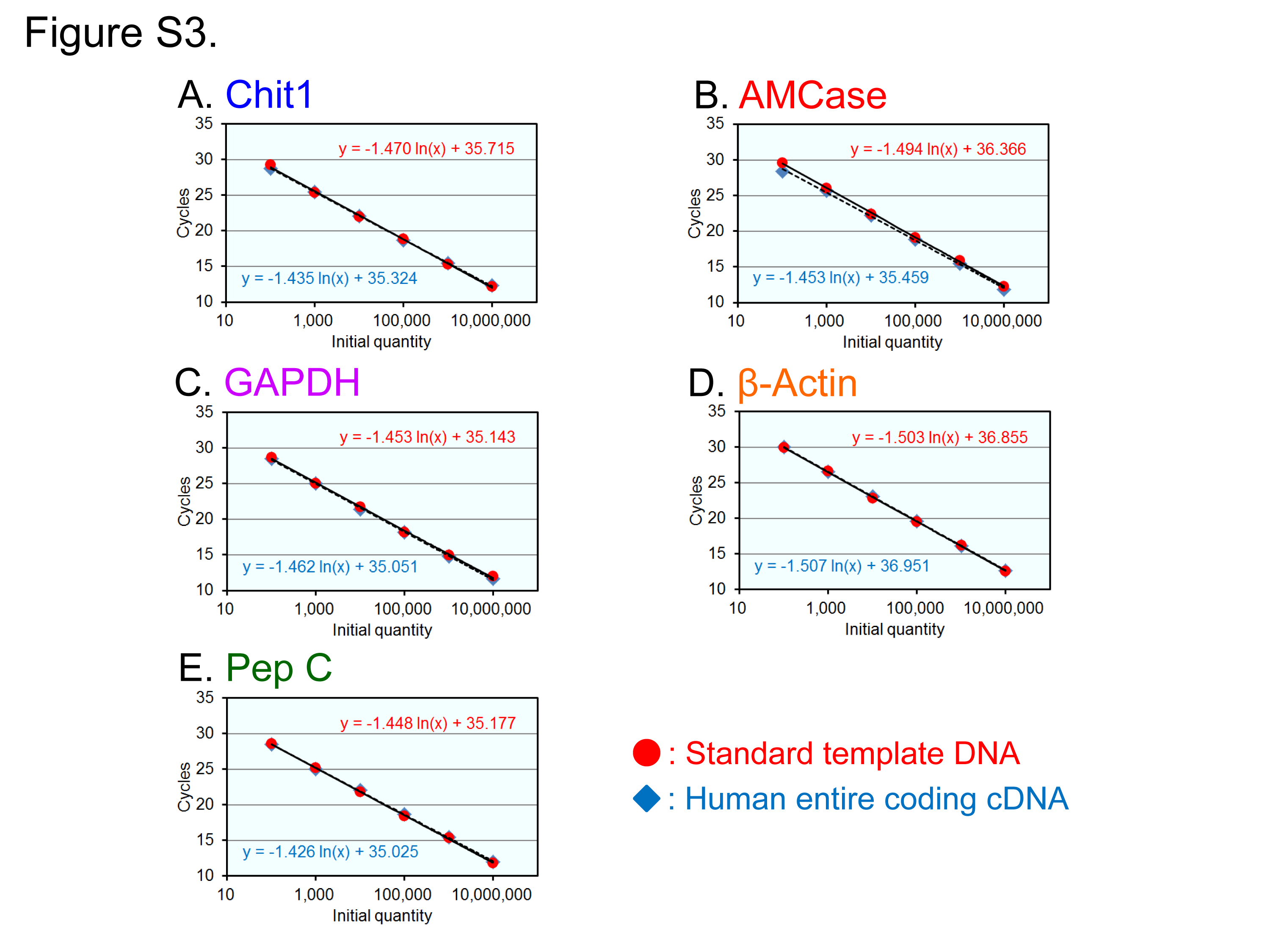

Supplement: Figure S3 — Development and validation of a quantitative real-time PCR system for the analysis of human tissues. The analyzed cDNAs were the following: A, Chit1; B, AMCase; C, GAPDH; D, β-actin; and E, pepsinogen C. Standard curves were obtained using the standard DNA containing the five human cDNA fragments (red closed circles). In addition, the quantification of the human entire coding cDNA was performed using the primer pairs for each gene. The target cDNA was amplified from a dilution of the entire coding cDNA with a known concentration and subsequently analyzed as an unknown sample (blue closed rhombuses). Equal quantities were obtained for each tested dilution of the standard curve and entire coding cDNA. (TIF) [file pone.0067399.s003.tif]

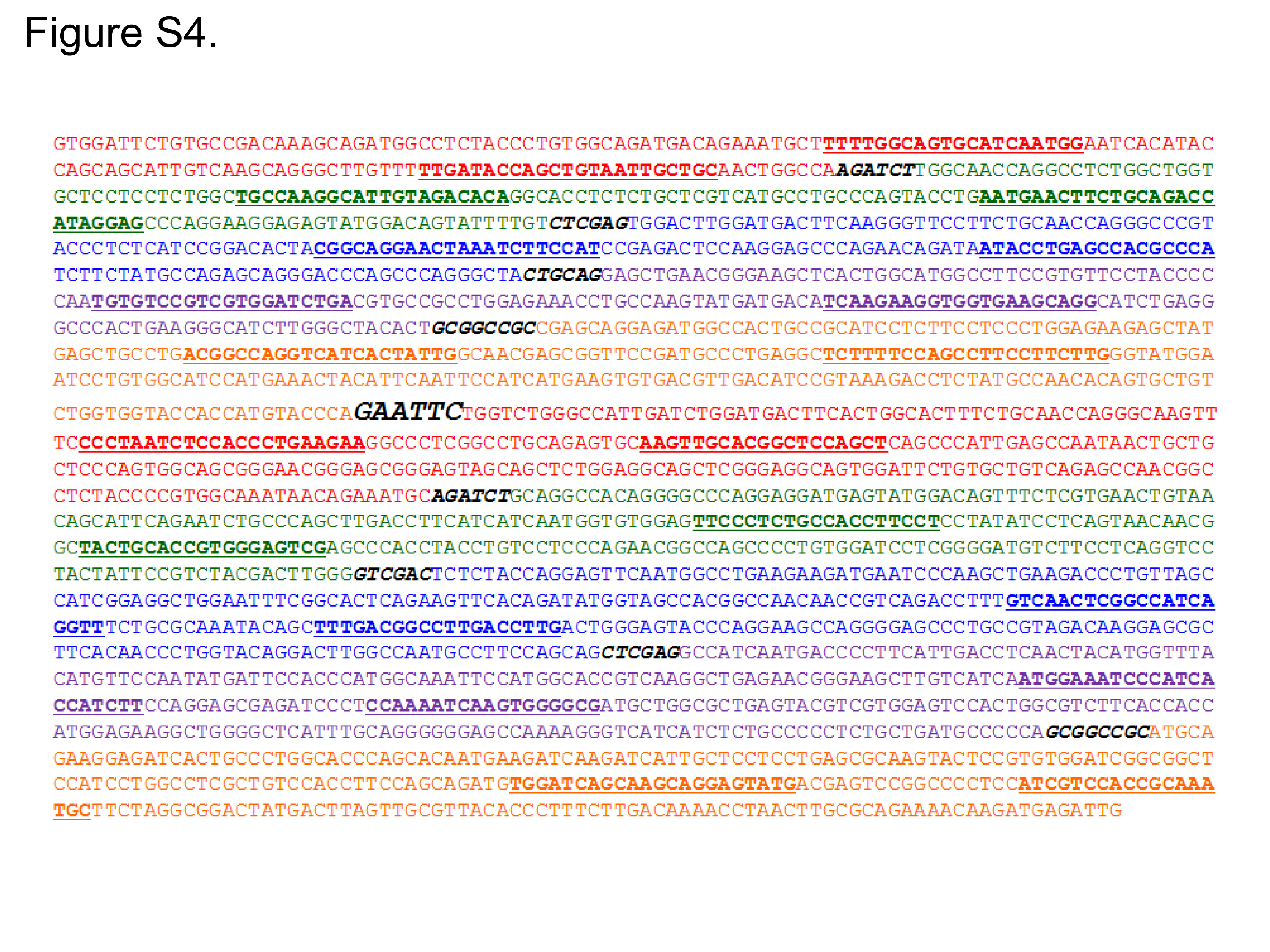

Supplement: Figure S4 — Nucleotide sequence of the human-mouse hybrid standard DNA. The human and mouse standard DNAs were ligated using the EcoRI site (shown in larger font, bold and italics) at a one-to-one ratio into a DNA fragment that was subsequently used as the human-mouse hybrid standard DNA. The 2,305-nucleotide-long DNA contained ten cDNA fragments (shown in different colors) that covered the PCR target regions (shown in bold and underlined) and 9–143 bases of the flanking regions of the human and mouse genes; it contained the appropriate restriction sites (shown in bold and italics). (TIF) [file pone.0067399.s004.tif]

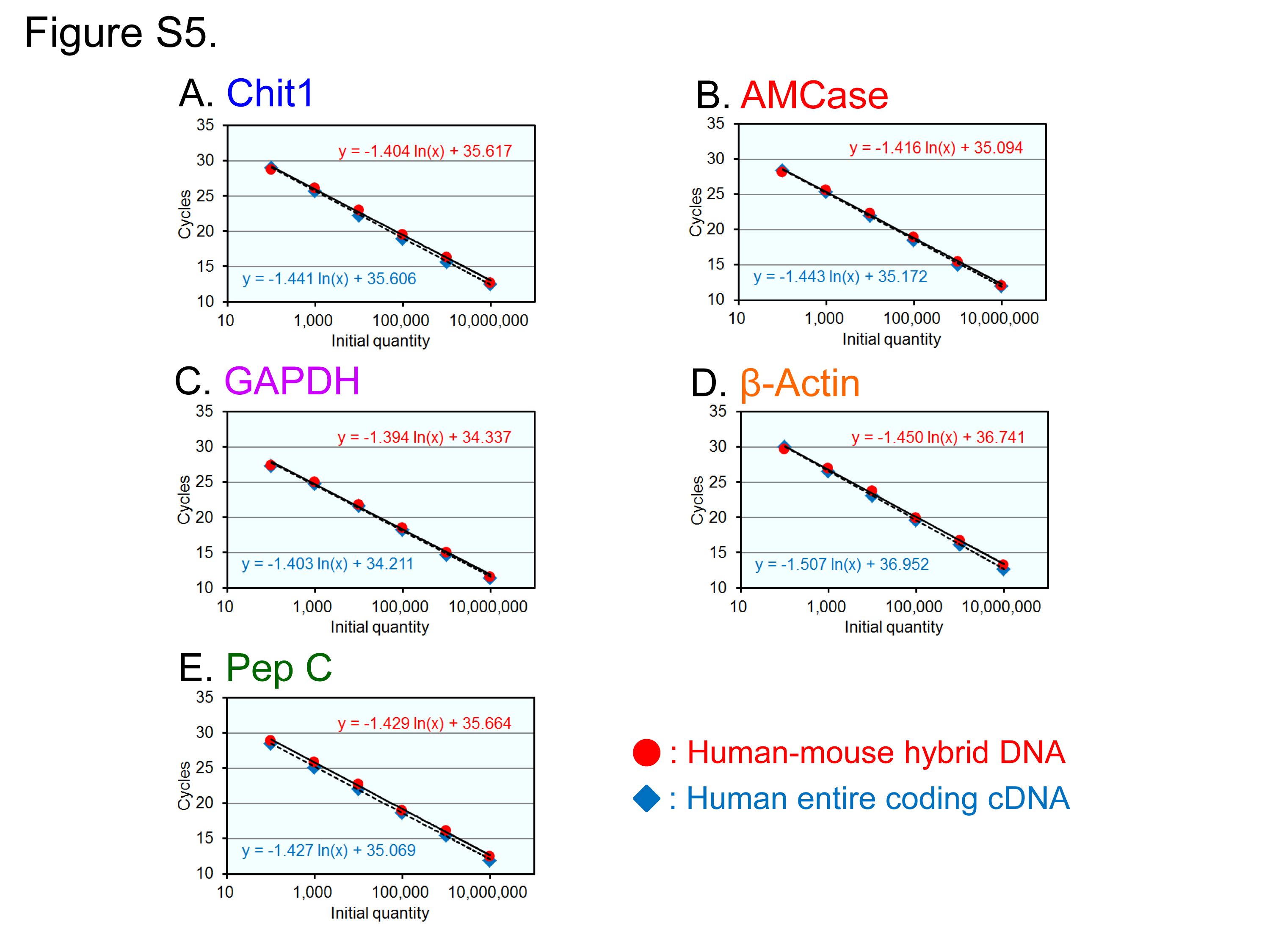

Supplement: Figure S5 — Development and validation of a real-time PCR system using the human-mouse hybrid standard DNA for the analysis of human genes. The analyzed human DNAs were the following: A, Chit1; B, AMCase; C, GAPDH; D, β-actin; and E, pepsinogen C. The standard curves were obtained using the hybrid standard DNA containing the five human cDNA fragments (red closed circles). In addition, the quantification of the human entire coding cDNAs was performed using primer pairs for each gene. The target cDNA was amplified from a dilution of the entire coding cDNA with a known concentration (see Figure S8) and subsequently analyzed as an unknown sample (blue closed rhombuses). Equal quantities were observed for each tested dilution of the standard curve and entire coding cDNA. (TIF) [file pone.0067399.s005.tif]

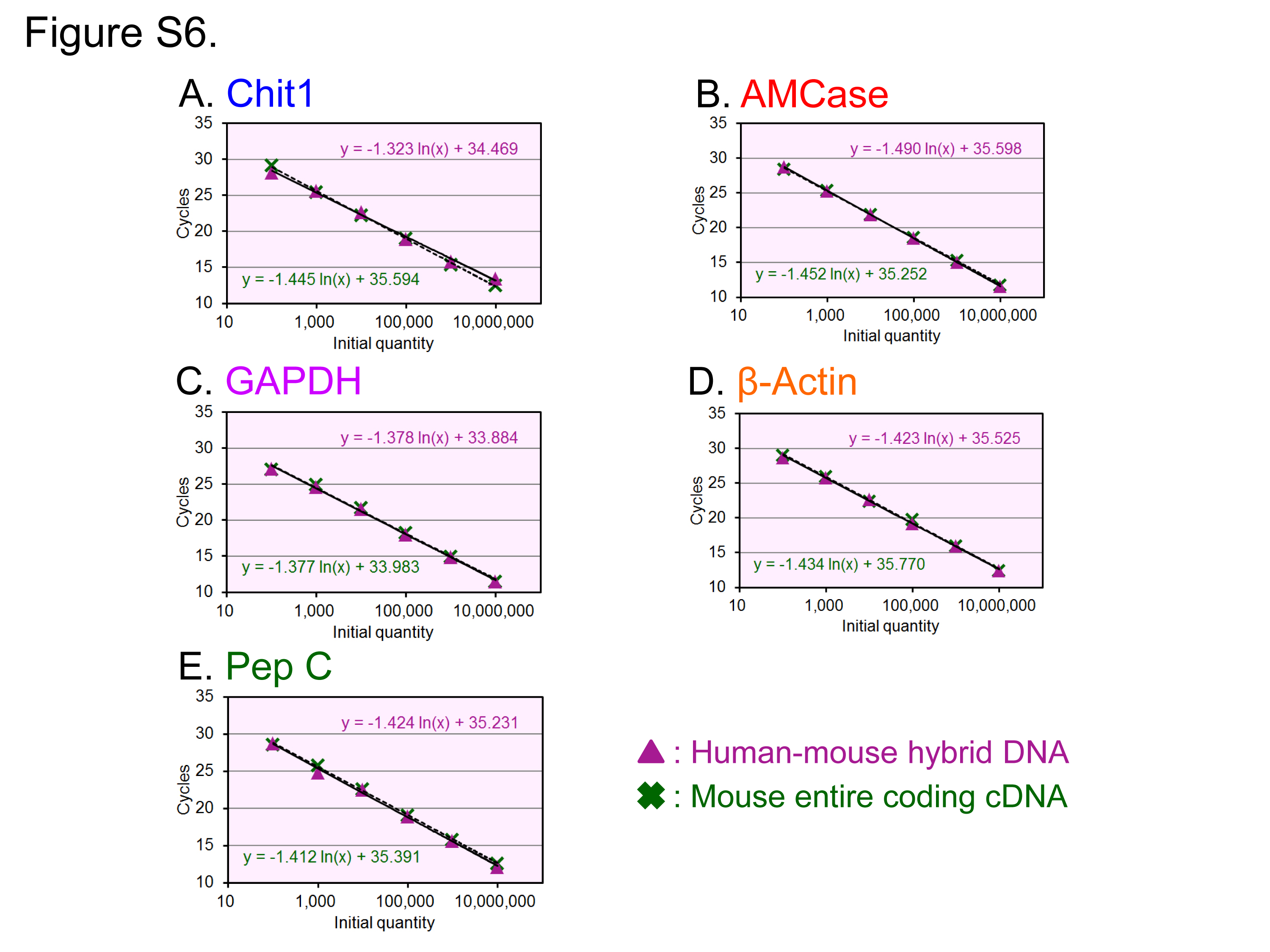

Supplement: Figure S6 — Development and validation of a real-time PCR system using the human-mouse hybrid standard DNA for the analysis of mouse genes. The same experiments as those shown in Figure S5 were performed for the mouse genes. The standard curves were obtained using the hybrid standard DNA containing the five mouse cDNA fragments (purple closed triangles). In addition, the quantification of the mouse entire coding cDNAs was performed using primer pairs for each gene. The target cDNA was amplified from a dilution of the entire coding cDNA with a known concentration and subsequently analyzed as an unknown sample (green crosses). Equal quantities were observed for each tested dilution of the standard curve and entire coding cDNA. (TIF) [file pone.0067399.s006.tif]

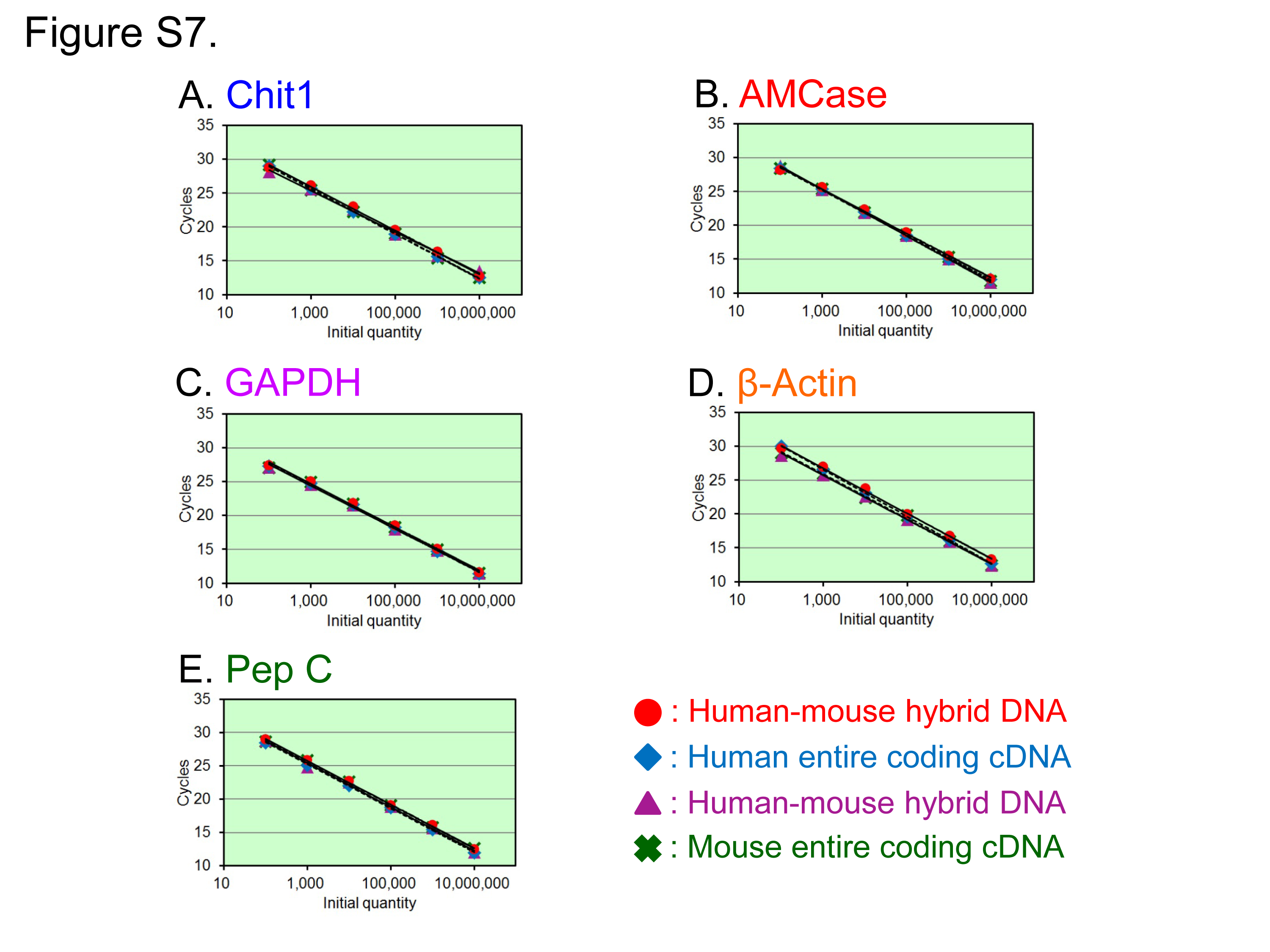

Supplement: Figure S7 — Development and validation of a quantitative real-time PCR system using the standard DNA. The results in Figure S5 and Figure S6 were superimposed. The quantification of low-abundance and abundant human and mouse transcripts allowed validation of the sensitivity and reliability of the real-time PCR system. The results indicate that our real-time PCR system and the human-mouse hybrid standard DNA offer a large dynamic quantification range that exhibits high accuracy and high sensitivity. (TIF) [file pone.0067399.s007.tif]
