# Supplementary material for: Quantification of Chitinase mRNA Levels in Human and Mouse Tissues by Real-Time PCR: Species-Specific Expression of Acidic Mammalian Chitinase in Stomach Tissues
Source: PLoS One. 2013 Jun 27;8(6):e67399. doi: 10.1371/journal.pone.0067399 (PMC3694897; doi:10.1371/journal.pone.0067399)
Supplement: Figure S8 — Nucleotide sequence and calculated molecular weight of the human entire coding cDNAs. (DOC) [file pone.0067399.s008.doc]

**Human Chit1**

**MW 908,917.4**

CATGGAATTCGGACCTGGAAAGCTGGTTTGTATGGGCTGCAGCCTGCCGCTGAGCTGCATCATGGTGCGGTCTGTGGCCTGGGCAGGTTTCATGGTCCTGCTGATGATCCCATGGGGCTCTGCTGCAAAACTGGTCTGCTACTTCACCAACTGGGCCCAGTACAGACAGGGGGAGGCTCGCTTCCTGCCCAAGGACTTGGACCCCAGCCTTTGCACCCACCTCATCTACGCCTTCGCTGGCATGACCAACCACCAGCTGAGCACCACTGAGTGGAATGACGAGACTCTCTACCAGGAGTTCAATGGCCTGAAGAAGATGAATCCCAAGCTGAAGACCCTGTTAGCCATCGGAGGCTGGAATTTCGGCACTCAGAAGTTCACAGATATGGTAGCCACGGCCAACAACCGTCAGACCTTTGTCAACTCGGCCATCAGGTTTCTGCGCAAATACAGCTTTGACGGCCTTGACCTTGACTGGGAGTACCCAGGAAGCCAGGGGAGCCCTGCCGTAGACAAGGAGCGCTTCACAACCCTGGTACAGGACTTGGCCAATGCCTTCCAGCAGGAAGCCCAGACCTCAGGGAAGGAACGCCTTCTTCTGAGTGCAGCGGTTCCAGCTGGGCAGACCTATGTGGATGCTGGATACGAGGTGGACAAAATCGCCCAGAACCTGGATTTTGTCAACCTTATGGCCTACGACTTCCATGGCTCTTGGGAGAAGGTCACGGGACATAACAGCCCCCTCTACAAGAGGCAAGAAGAGAGTGGTGCAGCAGCCAGCCTCAACGTGGATGCTGCTGTGCAACAGTGGCTGCAGAAGGGGACCCCTGCCAGCAAGCTGATCCTTGGCATGCCTACCTACGGACGCTCCTTCACACTGGCCTCCTCATCAGACACCAGAGTGGGGGCCCCAGCCACAGGGTCTGGCACTCCAGGCCCCTTCACCAAGGAAGGAGGGATGCTGGCCTACTATGAAGTCTGCTCCTGGAAGGGGGCCACCAAACAGAGAATCCAGGATCAGAAGGTGCCCTACATCTTCCGGGACAACCAGTGGGTGGGCTTTGATGATGTGGAGAGCTTCAAAACCAAGGTCAGCTATCTGAAGCAGAAGGGACTGGGCGGGGCCATGGTCTGGGCACTGGACTTAGATGACTTTGCCGGCTTCTCCTGCAACCAGGGCCGATACCCCCTCATCCAGACGCTACGGCAGGAACTGAGTCTTCCATACTTGCCTTCAGGCACCCCAGAGCTTGAAGTTCCAAAACCAGGTCAGCCCTCTGAACCTGAGCATGGCCCCAGCCCTGGACAAGACACGTTCTGCCAGGGCAAAGCTGATGGGCTCTATCCCAATCCTCGGGAACGGTCCAGCTTCTACAGCTGTGCAGCGGGGCGGCTGTTCCAGCAAAGCTGCCCGACAGGCCTGGTGTTCAGCAACTCCTGCAAATGCTGCACCTGGAATgctcgaggtcac

**Human AMCase**

**MW 899,602.4**

GCTACGGAATTCAACCATGACAAAGCTTATTCTCCTCACAGGTCTTGTCCTTATACTGAATTTGCAGCTCGGCTCTGCCTACCAGCTGACATGCTACTTCACCAACTGGGCCCAGTACCGGCCAGGCCTGGGGCGCTTCATGCCTGACAACATCGACCCCTGCCTCTGTACCCACCTGATCTACGCCTTTGCTGGGAGGCAGAACAACGAGATCACCACCATCGAATGGAATGATGTGACTCTCTACCAAGCTTTCAATGGCCTGAAAAATAAGAACAGCCAGCTGAAAACTCTCCTGGCCATTGGAGGCTGGAACTTCGGGACTGCCCCTTTCACTGCCATGGTTTCTACTCCTGAGAACCGCCAGACTTTCATCACCTCAGTCATCAAATTCCTGCGCCAGTATGAGTTTGACGGGCTGGACTTTGACTGGGAGTACCCTGGCTCTCGTGGGAGCCCTCCTCAGGACAAGCATCTCTTCACTGTCCTGGTGCAGGAAATGCGTGAAGCTTTTGAGCAGGAGGCCAAGCAGATCAACAAGCCCAGGCTGATGGTCACTGCTGCAGTAGCTGCTGGCATCTCCAATATCCAGTCTGGCTATGAGATCCCCCAACTGTCACAGTACCTGGACTACATCCATGTCATGACCTACGACCTCCATGGCTCCTGGGAGGGCTACACTGGAGAGAACAGCCCCCTCTACAAATACCCGACTGACACCGGCAGCAACGCCTACCTCAATGTGGATTATGTCATGAACTACTGGAAGGACAATGGAGCACCAGCTGAGAAGCTCATCGTTGGATTCCCTACCTATGGACACAACTTCATCCTGAGCAACCCCTCCAACACTGGAATTGGTGCCCCCACCTCTGGTGCTGGTCCTGCTGGGCCCTATGCCAAGGAGTCTGGGATCTGGGCTTACTACGAGATCTGTACCTTCCTGAAAAATGGAGCCACTCAGGGATGGGATGCCCCTCAGGAAGTGCCTTATGCCTATCAGGGCAATGTGTGGGTTGGCTATGACAACATCAAGAGCTTCGATATTAAGGCTCAATGGCTTAAGCACAACAAATTTGGAGGCGCCATGGTCTGGGCCATTGATCTGGATGACTTCACTGGCACTTTCTGCAACCAGGGCAAGTTTCCCCTAATCTCCACCCTGAAGAAGGCCCTCGGCCTGCAGAGTGCAAGTTGCACGGCTCCAGCTCAGCCCATTGAGCCAATAACTGCTGCTCCCAGTGGCAGCGGGAACGGGAGCGGGAGTAGCAGCTCTGGAGGCAGCTCGGGAGGCAGTGGATTCTGTGCTGTCAGAGCCAACGGCCTCTACCCCGTGGCAAATAACAGAAATGCCTTCTGGCACTGCGTGAATGGAGTCACGTACCAGCAGAACTGCCAGGCCGGGCTTGTCTTCGACACCAGCTGTGATTGCTGCAACTGGGCAGCTCGAGGTCAC

**Human GAPDH**

**MW 624,009.0**

CCATGGGGAAGGTGAAGGTCGGAGTCAACGGATTTGGTCGTATTGGGCGCCTGGTCACCAGGGCTGCTTTTAACTCTGGTAAAGTGGATATTGTTGCCATCAATGACCCCTTCATTGACCTCAACTACATGGTTTACATGTTCCAATATGATTCCACCCATGGCAAATTCCATGGCACCGTCAAGGCTGAGAACGGGAAGCTTGTCATCAATGGAAATCCCATCACCATCTTCCAGGAGCGAGATCCCTCCAAAATCAAGTGGGGCGATGCTGGCGCTGAGTACGTCGTGGAGTCCACTGGCGTCTTCACCACCATGGAGAAGGCTGGGGCTCATTTGCAGGGGGGAGCCAAAAGGGTCATCATCTCTGCCCCCTCTGCTGATGCCCCCATGTTCGTCATGGGTGTGAACCATGAGAAGTATGACAACAGCCTCAAGATCATCAGCAATGCCTCCTGCACCACCAACTGCTTAGCACCCCTGGCCAAGGTCATCCATGACAACTTTGGTATCGTGGAAGGACTCATGACCACAGTCCATGCCATCACTGCCACCCAGAAGACTGTGGATGGCCCCTCCGGGAAACTGTGGCGTGATGGCCGCGGGGCTCTCCAGAACATCATCCCTGCCTCTACTGGCGCTGCCAAGGCTGTGGGCAAGGTCATCCCTGAGCTGAACGGGAAGCTCACTGGCATGGCCTTCCGTGTCCCCACTGCCAACGTGTCAGTGGTGGACCTGACCTGCCGTCTAGAAAAACCTGCCAAATATGATGACATCAAGAAGGTGGTGAAGCAGGCGTCGGAGGGCCCCCTCAAGGGCATCCTGGGCTACACTGAGCACCAGGTGGTCTCCTCTGACTTCAACAGCGACACCCACTCCTCCACCTTTGACGCTGGGGCTGGCATTGCCCTCAACGACCACTTTGTCAAGCTCATTTCCTGGTATGACAACGAATTTGGCTACAGCAACAGGGTGGTGGACCTCATGGCCCACATGGCCTCCAAGGAGTAA

**Human β-actin**

**MW 701,314.0**

CACCATGGATGATGATATCGCCGCGCTCGTCGTCGACAACGGCTCCGGCATGTGCAAGGCCGGCTTCGCGGGCGACGATGCCCCCCGGGCCGTCTTCCCCTCCATCGTGGGGCGCCCCAGGCACCAGGGCGTGATGGTGGGCATGGGTCAGAAGGATTCCTATGTGGGCGACGAGGCCCAGAGCAAGAGAGGCATCCTCACCCTGAAGTACCCCATCGAGCACGGCATCGTCACCAACTGGGACGACATGGAGAAAATCTGGCACCACACCTTCTACAATGAGCTGCGTGTGGCTCCCGAGGAGCACCCCGTGCTGCTGACCGAGGCCCCCCTGAACCCCAAGGCCAACCGCGAGAAGATGACCCAGATCATGTTTGAGACCTTCAACACCCCAGCCATGTACGTTGCTATCCAGGCTGTGCTATCCCTGTACGCCTCTGGCCGTACCACTGGCATCGTGATGGACTCCGGTGACGGGGTCACCCACACTGTGCCCATCTACGAGGGGTATGCCCTCCCCCATGCCATCCTGCGTCTGGACCTGGCTGGCCGGGACCTGACTGACTACCTCATGAAGATCCTCACCGAGCGCGGCTACAGCTTCACCACCACGGCCGAGCGGGAAATCGTGCGTGACATTAAGGAGAAGCTGTGCTACGTCGCCCTGGACTTCGAGCAAGAGATGGCCACGGCTGCTTCCAGCTCCTCCCTGGAGAAGAGCTACGAGCTGCCTGACGGCCAGGTCATCACCATTGGCAATGAGCGGTTCCGCTGCCCTGAGGCACTCTTCCAGCCTTCCTTCCTGGGCATGGAGTCCTGTGGCATCCACGAAACTACCTTCAACTCCATCATGAAGTGTGACGTGGACATCCGCAAAGACCTGTACGCCAACACAGTGCTGTCTGGCGGCACCACCATGTACCCTGGCATTGCCGACAGGATGCAGAAGGAGATCACTGCCCTGGCACCCAGCACAATGAAGATCAAGATCATTGCTCCTCCTGAGCGCAAGTACTCCGTGTGGATCGGCGGCTCCATCCTGGCCTCGCTGTCCACCTTCCAGCAGATGTGGATCAGCAAGCAGGAGTATGACGAGTCCGGCCCCTCCATCGTCCACCGCAAATGCTTCTAGGCG

**Human Pepsinogen C**

**MW 738,363.0**

CTCTGTGGCCAGTTGGGGACCAGCATCATGAAGTGGATGGTGGTGGTCTTGGTCTGCCTCCAGCTCTTGGAGGCAGCAGTGGTCAAAGTGCCCCTGAAGAAATTTAAGTCTATCCGTGAGACCATGAAGGAGAAGGGCTTGCTGGGGGAGTTCCTGAGGACCCACAAGTATGATCCTGCTTGGAAGTACCGCTTTGGTGACCTCAGCGTGACCTACGAGCCCATGGCCTACATGGATGCTGCCTACTTTGGTGAGATCAGCATCGGGACTCCACCCCAGAACTTCCTGGTCCTTTTTGACACCGGCTCCTCCAACTTGTGGGTGCCCTCTGTCTACTGCCAGAGCCAGGCCTGCACCAGTCACTCCCGCTTCAACCCCAGCGAGTCGTCCACCTACTCCACCAATGGGCAGACCTTCTCCCTGCAGTATGGCAGTGGCAGCCTCACCGGCTTCTTTGGCTATGACACCCTGACTGTCCAGAGCATCCAGGTCCCCAACCAGGAGTTCGGCTTGAGTGAGAATGAGCCTGGTACCAACTTCGTCTATGCGCAGTTTGATGGCATCATGGGCCTGGCCTACCCTGCTCTGTCCGTGGATGAGGCCACCACAGCTATGCAGGGCATGGTGCAGGAGGGCGCCCTCACCAGCCCCGTCTTCAGCGTCTACCTCAGCAACCAGCAGGGCTCCAGCGGGGGAGCGGTTGTCTTTGGGGGTGTGGATAGCAGCCTGTACACGGGGCAGATCTACTGGGCGCCTGTCACCCAGGAACTCTACTGGCAGATTGGCATTGAAGAGTTCCTCATCGGCGGCCAGGCCTCCGGCTGGTGTTCTGAGGGTTGCCAGGCCATCGTGGACACAGGCACCTCTCTGCTCACTGTGCCCCAGCAGTACATGAGTGCTCTTCTGCAGGCCACAGGGGCCCAGGAGGATGAGTATGGACAGTTTCTCGTGAACTGTAACAGCATTCAGAATCTGCCCAGCTTGACCTTCATCATCAATGGTGTGGAGTTCCCTCTGCCACCTTCCTCCTATATCCTCAGTAACAACGGCTACTGCACCGTGGGAGTCGAGCCCACCTACCTGTCCTCCCAGAACGGCCAGCCCCTGTGGATCCTCGGGGATGTCTTCCTCAGGTCCTACTATTCCGTCTACGACTTGGGCAACAACAGAGTAGGCTTTGCCACTGCCGCCTAGA
