# Supplementary material for: Quantification of Chitinase mRNA Levels in Human and Mouse Tissues by Real-Time PCR: Species-Specific Expression of Acidic Mammalian Chitinase in Stomach Tissues
Source: PLoS One. 2013 Jun 27;8(6):e67399. doi: 10.1371/journal.pone.0067399 (PMC3694897; doi:10.1371/journal.pone.0067399)
Supplement: Table S1 — The nucleotide sequences of the primers that were selected for the real-time PCR for the human system. (DOC) [file pone.0067399.s009.doc]

H_Chit1_Fw: GTCAACTCGGCCATCAGGTT

H_Chit1_Rv: CAAGGTCAAGGCCGTCAAA

H_AMCase_Fw: CCCTAATCTCCACCCTGAAGAA

H_AMCase_Rv: AGCTGGAGCCGTGCAACTT

H_GAPDH_Fw: ATGGAAATCCCATCACCATCTT

H_GAPDH_Rv: CGCCCCACTTGATTTTGG

H_β-Actin_Fw: TGGATCAGCAAGCAGGAGTATG

H_β-Actin_Rv: GCATTTGCGGTGGACGAT

H_Pep C_Fw: TTCCCTCTGCCACCTTCCT

H_Pep C_Rv: CGACTCCCACGGTGCAGTA
