# Supplementary material for: Quantification of Chitinase mRNA Levels in Human and Mouse Tissues by Real-Time PCR: Species-Specific Expression of Acidic Mammalian Chitinase in Stomach Tissues
Source: PLoS One. 2013 Jun 27;8(6):e67399. doi: 10.1371/journal.pone.0067399 (PMC3694897; doi:10.1371/journal.pone.0067399)
Supplement: Table S2 — Forward and reverse primers used to construct the human standard template DNA for real-time PCR. (DOC) [file pone.0067399.s010.doc]

Eco_quant_H_AMCase_Fw: CATGGAATTCTGGTCTGGGCCATTGATCTGGATGA

Bgl_quant_H_AMCase_Rv: TGACAGATCTGCATTTCTGTTATTTGCCACGGGGT

Bgl_quant_H_Pep C_Fw: TGACAGATCTGCAGGCCACAGGGGCCCAGGAGGAT

Sal_quant_H_Pep C_Rv: TGACGTCGACCCCAAGTCGTAGACGGAATAGTAGG

Sal_quant_H_Chit1_Fw: TGACGTCGACTCTCTACCAGGAGTTCAATGGCCTG

Xho_quant_H_Chit1_Rv: TGACCTCGAGCTGCTGGAAGGCATTGGCCAAGTCC

Xho_quant_H_GAPDH_Fw: TGACCTCGAGGCCATCAATGACCCCTTCATTGACC

Not_quant_H_GAPDH_Rv: TCGAGCGGCCGCTGGGGGCATCAGCAGAGGGGGCA

Not_quant_H_β-Actin_Fw: TCGAGCGGCCGCATGCAGAAGGAGATCACTGCCCT

Quant_H_β-Actin_Rv: CAATCTCATCTTGTTTTCTGCGCAAGTTAGG
