# Supplementary material for: Quantification of Chitinase mRNA Levels in Human and Mouse Tissues by Real-Time PCR: Species-Specific Expression of Acidic Mammalian Chitinase in Stomach Tissues
Source: PLoS One. 2013 Jun 27;8(6):e67399. doi: 10.1371/journal.pone.0067399 (PMC3694897; doi:10.1371/journal.pone.0067399)
Supplement: Table S3 — Primers for PCR amplification of the human entire coding cDNAs. (DOC) [file pone.0067399.s011.doc]

H_entire_Chit1_Fw: CATGGAATTCGGACCTGGAAAGCTGGTTTGTATGG

H_entire_Chit1_RV: GTGACCTCGAGCATTCCAGGTGCAGCATTTGCAGG

H_entire_AMCase_FW: GCTACGGAATTCAACCATGACAAAGCTTATTCTCC

H_entire_AMCase_RV: GTGACCTCGAGCTGCCCAGTTGCAGCAATCACAGC

H_entire_GAPDH_Fw: CCATGGGGAAGGTGAAGGTCGGAGT

H_entire_GAPDH_Rv: TTACTCCTTGGAGGCCATGTGGGCC

H_entire_β-Actin_Fw: CACCATGGATGATGATATCGCCGCG

H_entire_β-Actin_Rv: CGCCTAGAAGCATTTGCGGTGGACG

H_entire_Pep C_Fw: CTCTGTGGCCAGTTGGGGACCAGCA

H_entire_Pep C Rv: TCTAGGCGGCAGTGGCAAAGCCTAC
